# Fitness Measures Among Boy Scouts Completing the Personal Fitness Merit Badge

**DOI:** 10.7759/cureus.2538

**Published:** 2018-04-25

**Authors:** Sara Arena, Leah Riley, Gregory Schilz, Eric Schultz, Bethany Watterworth, Edward Peterson

**Affiliations:** 1 Physical Therapy, Oakland University; 2 Department of Public Health Science, Henry Ford Health System

**Keywords:** boy scouts, fitness testing, body mass index

## Abstract

Introduction

A formative framework for positive lifestyle and health behaviors is established during adolescence. The Boy Scouts of America (BSA) is one organization that promotes healthy lifestyles among adolescent males and includes earning the Personal Fitness Merit Badge (PFMB). Despite the BSA Oath “to keep myself physically strong”, there is a paucity of evidence describing fitness test (FT) outcomes or correlations between FT and variables including age or body mass index (BMI) among Boy Scouts. Therefore, the purpose of this study is to describe and correlate FT to age and BMI among adolescent Boy Scouts.

Methods

A prospective study design recruited Boy Scouts aged 11-17 years from Southeast Michigan using a sample of convenience. After securing physician clearance to participate, FT measures were performed utilizing previously established methodology. Third year doctor of physical therapy students trained in the measurement protocol performed the FT measures. Measures encompassed those required for the PFMB: BMI, pull-ups, push-ups, sit and reach flexibility test (SRF), sit-ups, and the one mile run (1MR). Data was analyzed using descriptive statistics, a Kruskal-Wallis test examined relationships between BMI and FT, and a nonparametric Spearman correlation examined correlations between FT performance and both age and BMI with statistical significance set at less than 0.05.

Results

Ninety-nine Boy Scouts, whose mean age was 12.6 years (SD 1.4), met the inclusion criteria. The mean BMI was 21.5 (kg/height (cm)) ^2 ^(SD 5.4) with eight scouts meeting criteria of underweight, 56 normal weight, 14 overweight, and 21 obese. Fitness test results were as follows: pull-ups 1.75 (SD 2.7), push-ups 18.0 (SD 10.6), SRF 21.2 cm (SD 10.5), sit-ups 28.9 (SD 8.7), and the 1MR run 616.5 seconds (SD 156.8). When comparing normal weight scouts to overweight and obese scouts, a significantly higher frequency of pull-ups (p=0.002, p=.001), push-ups (p=0.02, p=0.03), sit-ups (p=0.01, p=0.003,), and decreased time for completion on the 1MR (p=0.001, p=0.001) was identified, respectively. Furthermore, while no correlations were identified by age, a negative correlation was identified between increased sit-up frequency (r=-0.36, p=0.001) and decreased 1MR time performance (r=0.39, p=0.001) and a higher BMI.

Discussion

While prior evidence suggests improved FT scores in adolescent males with advancing age, this was not observed. Less favorable PFMB required FT performance with an increased BMI among Boy Scouts is in congruence with prior reports for adolescent males.

Conclusion

Variations in FT performance levels were observed among Boy Scouts completing the initial FT requirements of the PFMB. However, correlations between higher BMI and less favorable FT performance were detected.

## Introduction

Physical activity is important in the adolescent years to reduce cardiovascular risk factors and improve overall fitness [1-3]. A quadrupling of obesity rates over the past three decades [4] emphasizes the need for interventions targeted toward thwarting this heath risk. The Boy Scouts of America (BSA) is a national organization that promotes healthy lifestyle choices among adolescent boys. It offers a Personal Fitness Merit Badge (PFMB) in support of their oath which states that scouts are to keep themselves “physically strong” [5]. Furthermore, this merit badge is a prerequisite for achieving the rank of Eagle [6], which is the highest youth rank within the BSA.

Esmaeilzadeh et al. examined differences in physical fitness and sedentary activity levels among 7- to 11-year-old boys with varied body mass index (BMI) categories [3]. The study found that as BMI increased, physical fitness activities decreased and sedentary activities increased. While studies specific to the physical fitness levels of Boy Scouts are limited, a study by Jago et al. [1] examined physical activity interventions on scouts. The authors reported that exercise sessions conducted during the weekly troop meetings did result in positive change toward increased physical activity and decreased sedentary activity; however, no significant change to BMI was observed. Furthermore, Kanehisa et al. [7] examined the influence of age on the development of strength and muscle cross-sectional area in 7- to 18-year-old males and reported that boys in early stages of puberty had reduced development of strength production with increased muscle cross-sectional area. It provided evidence to support increase in muscle development among males between 13 and 18 years when compared to younger male age groups.

Physical fitness is important during adolescence as it can be a predictor of future health status [2-4]. However, there is a paucity of evidence as to the fitness levels of Boy Scouts and the underlying health indicators that may contribute to more positive fitness test (FT) measures. A more thorough understanding of correlative relationships between a scout’s age or BMI and the various components of fitness may guide a scout’s successful completion of the PFMB. Furthermore, this knowledge may be of benefit when scouts design the individualized 12-week exercise plan required for the PFMB to address their unique health and wellness needs. Therefore, the purpose of this study is to describe and correlate FT to age and BMI among adolescent Boy Scouts.

## Materials and methods

Research design

After securing the Oakland University Institutional Review Board (IRB) approval to assure that the rights of each participant were protected, a prospective analytical study design was initiated utilizing a sample of convenience.

Sampling criteria

An anticipated 100 Boy Scouts (33-36 per year of data collection) were invited to participate in one four-hour fitness event on the campus of Oakland University. An anticipated 100 participants would result in a test with .80 power, with a two-sided 0.05 adjusted alpha level, to detect an effect size of 0.74. The event was conducted as a component of service learning in a Doctor of Physical Therapy (DPT) circularly required Health Promotion and Wellness in Physical Therapy course. The course enrolls between 33-36 students per year. To maintain a one-to-one scout to student ratio for data collection, a three-year recruitment time frame was anticipated.

Registered Boy Scouts from the BSA Great Lakes Council servicing Southeast Michigan were invited to participate in one of three identical events offered in either November of 2014, 2015, or 2016. The authors acknowledge the one year span between each of the three data collection points. However, the merit badge requirements and method of data collection remained constant at each of the three data collection encounters to reduce potential bias introduced by the time lapse. The Boy Scouts were informed of the opportunity through council advertising and flyers distributed by local troop scoutmasters.

Scouts met inclusion criteria if they were of the male gender, a registered member of the BSA, and if parental or guardian permission was secured prior to the event date. Scouts were 11 to 17 years of age as this is the general age requirement for BSA participation. Additionally, each scout had prior approval of their troop scoutmaster to begin work on their PFMB, and the parent or guardian confirmed that physician medical clearance was conducted within the year of FT and exercise participation. Scouts who were not registered members of the BSA, were less than 11 or older than 18 years, did not assent to research participation, had previously completed the PFMB, or were absent from the FT day were excluded from the study.

Protocol

Data collection was conducted in conjunction with a PFMB service learning event and provided scouts an opportunity to complete eight of the nine PFMB requirements. Scouts were aided in developing a 12-week exercise plan [5] which scouts then carried out independent of the data collection day. As the goals and requirements of the PFMB parallel the goals and objectives of the physical therapy health promotion and wellness coursework, DPT students in their third professional year of a DPT program engaged in a service learning opportunity that was mutually beneficial to both the DPT students and the scouts.

Under the direction of the course instructor, who was also a certified BSA merit badge counselor for the PFMB, DPT students enrolled in the health promotion and wellness course were trained in all testing methodology. They were then assigned a Boy Scout for whom they conducted all FTs and measures. Specifically, the merit badge requires baseline fitness testing of (1) BMI, (2) sit and reach flexibility test (SRF), (3) push-ups, (4) pull-ups, (5) sit-ups, and (6) the one-mile run (1MR) [5]. Additionally, the non-merit badge required FT included (1) grip, tip pinch, and key pinch strength, (2) Illinois Agility T-Test (T-Test), and (3) vertical jump. All FTs were performed under the supervision of a licensed physical therapist. Detailed methodology for each FT is outlined below.

Merit badge required fitness tests

Methods for obtaining all PFMB required FT measures utilized the protocols set forth in the BSA PFMB [5]. All measurements were obtained from indoor testing environments to control the variability that may result from an outdoor testing environment.

Body Mass Index

Body mass index was calculated after obtaining a scout's weight and height. Weight was measured and recorded in kilograms (kg) utilizing a Detector® calibrated scale (Webb City, MO). Height was measured in centimeters (cm) with the scout's shoes removed and back against a height rod included in the design of the scale. Height was converted to meters (m) and BMI (kg/m^2^) was then calculated utilizing the formula: weight (kg) / height (m)^2^ [8]. Body mass index was further categorized as underweight, normal, overweight, or obese using classifications set forth by the Centers for Disease Control and Prevention [9] for age percentiles as follows: underweight was defined as less than the 5th percentile, normal weight between the 5th and 84th percentiles, overweight category between the 85th and 94th percentile, and the obese category was defined as greater than or equal to the 95th percentile.

Sit and Reach Flexibility Test

**Figure 1:**
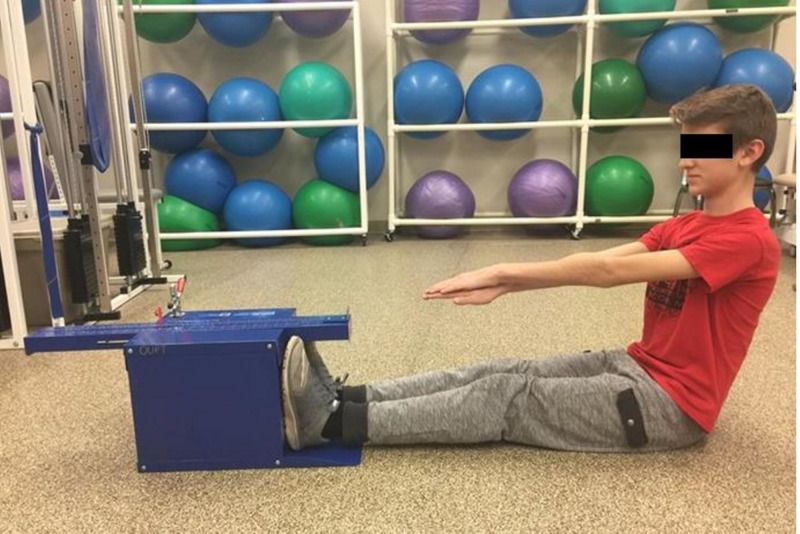
Sit and reach flexibility start position

**Figure 2:**
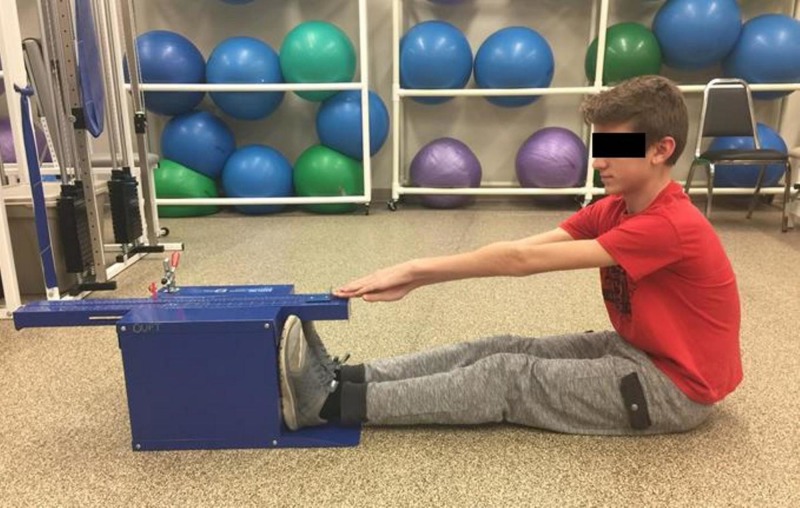
Sit and reach flexibility end position

Scouts were positioned in long sitting facing a Sit-and-Reach Flexibility Box (Baseline 12-1085, 4MD Medical Solutions Lakewood, NJ) with knees fully extended, posterior popliteal region flat on the floor, and feet flat against the end plate (Figure 1) as is the required measure of the PFMB [5]. The scout then stacked hands on top of each other with palms facing down and reached arms forward by flexing forward at the hips. The scouts extended along the top of the SRF measuring scale while holding their hands at a maximal outstretched position on the measuring scale. A total of three practice measurements were performed with a fourth and final reach recorded (Figure 2). Investigators monitored for rounding of shoulders and thorax to optimize accuracy of the lower extremity flexibility measures obtained.

Push-ups

**Figure 3:**
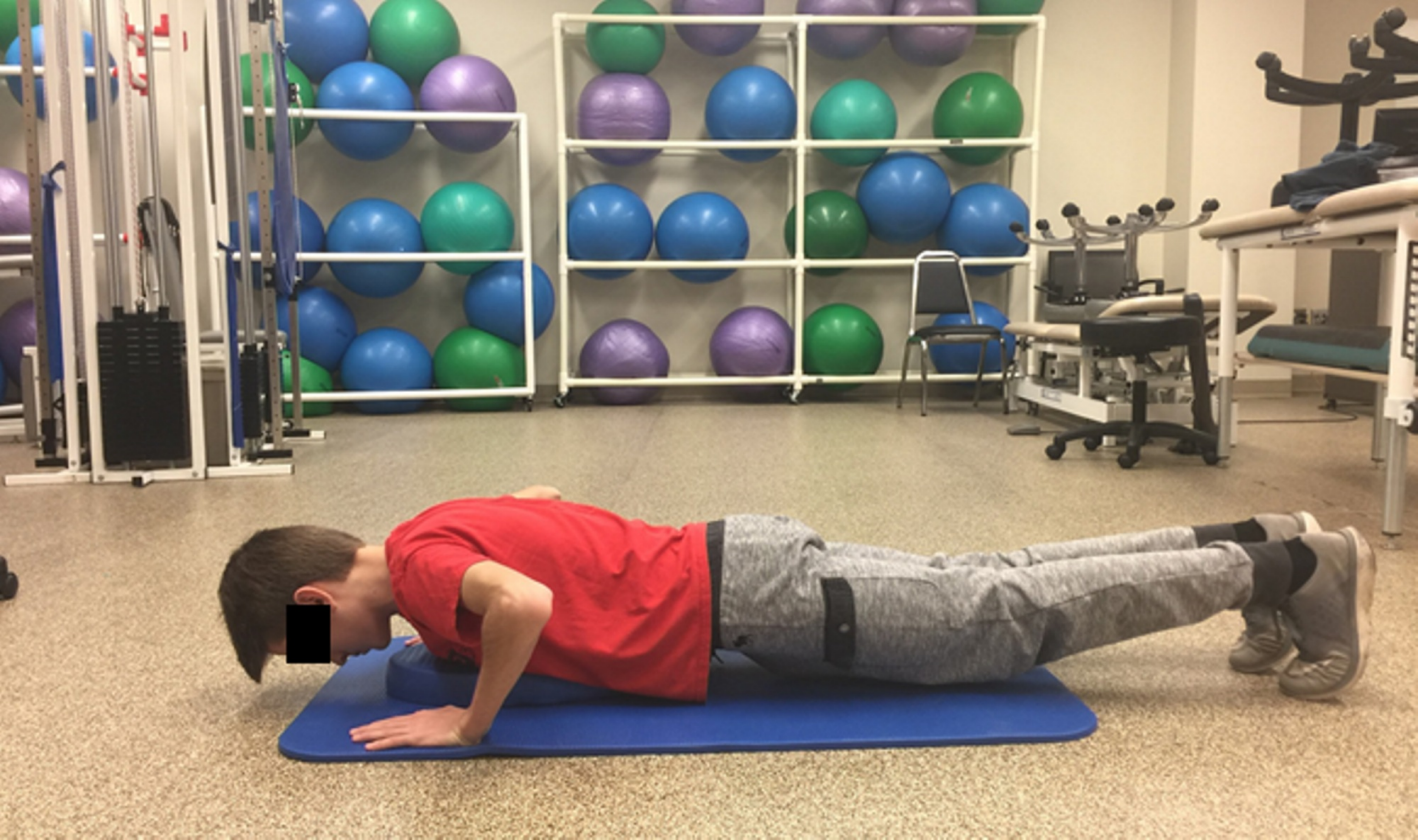
Push-up start position

**Figure 4:**
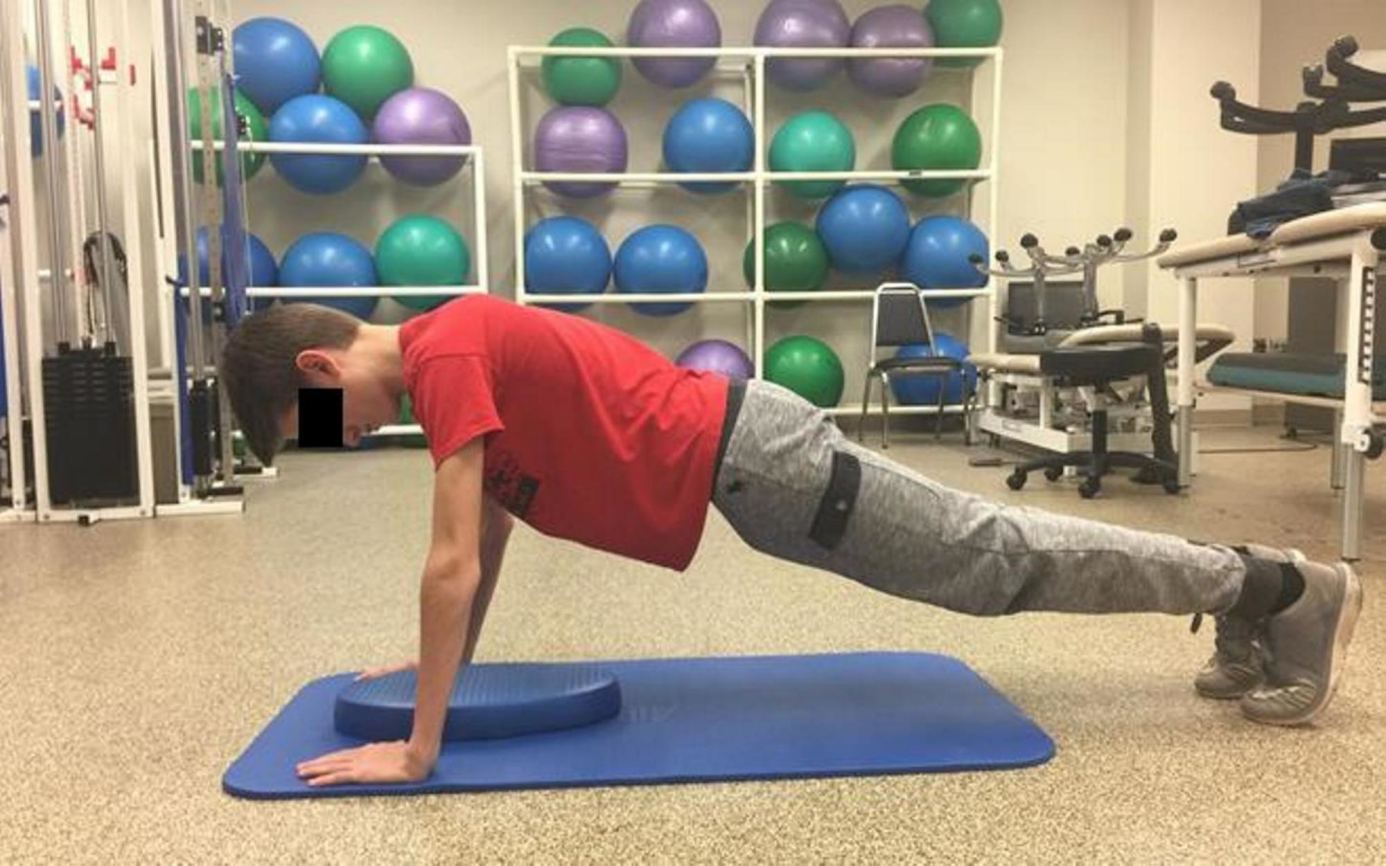
Push-up end position

A scout started in a prone position with palms placed flat on the floor under shoulders, feet in flexed position with ball of the foot and toes on the floor, and shoulders, hips, and legs in a straight line from the scout’s heels to the head (Figure 3) as is the required measurement protocol of the PFMB [5]. The scout pushed up by extending both arms, but avoiding full locked extension at elbows (Figure 4) and then lowered his body to a guide placed approximately 2 inches from the floor and directly under the scout’s chest. Scouts were instructed to complete as many repetitions as possible in 60 seconds.

Pull-ups

**Figure 5:**
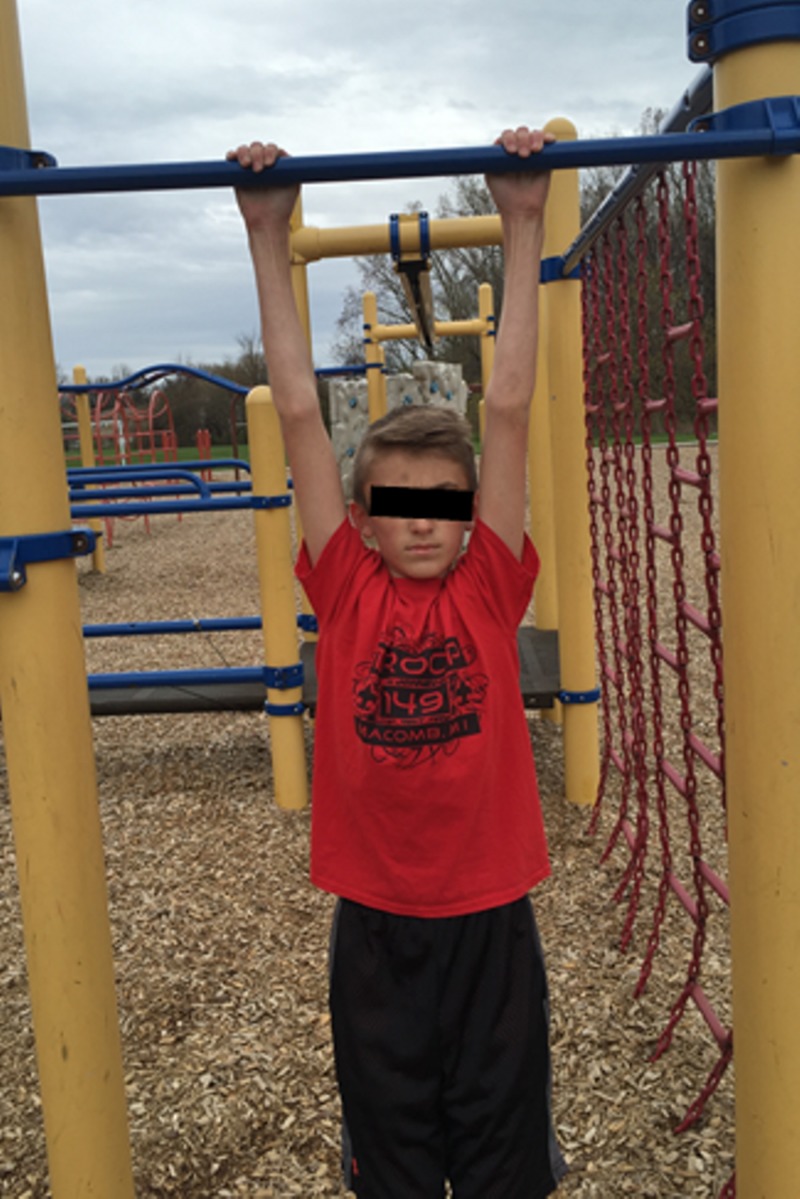
Pull-up start and end position

**Figure 6:**
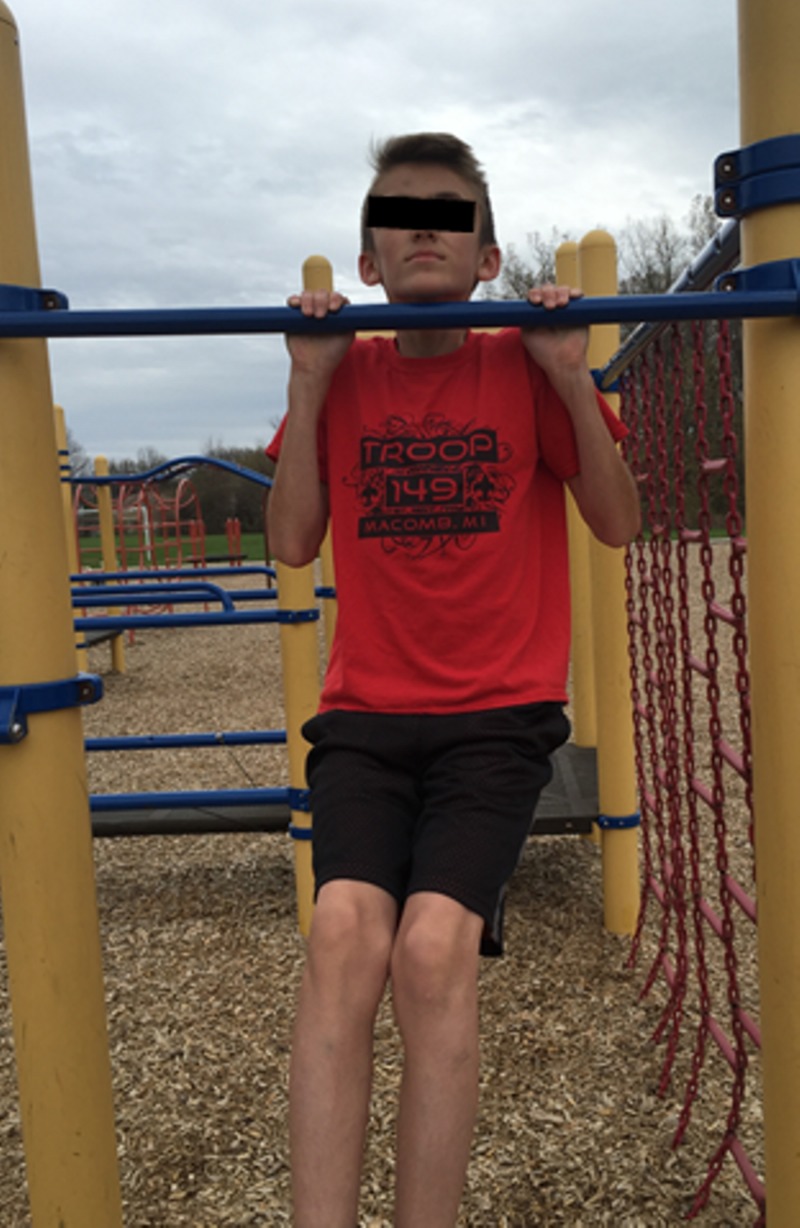
Pull-up mid-test position

Scouts began the exercise by hanging from a bar with arms fully extended, palms forward and directly above the shoulders (Figure 5) as is the required measurement protocol of the PFMB [5]. Scouts then pulled up until they touched the top of the bar with the bottom of an outstretched chin (Figure 6). The scout returned to the starting position and repeated the process as many times as possible in 60 seconds.

Sit-ups

**Figure 7:**
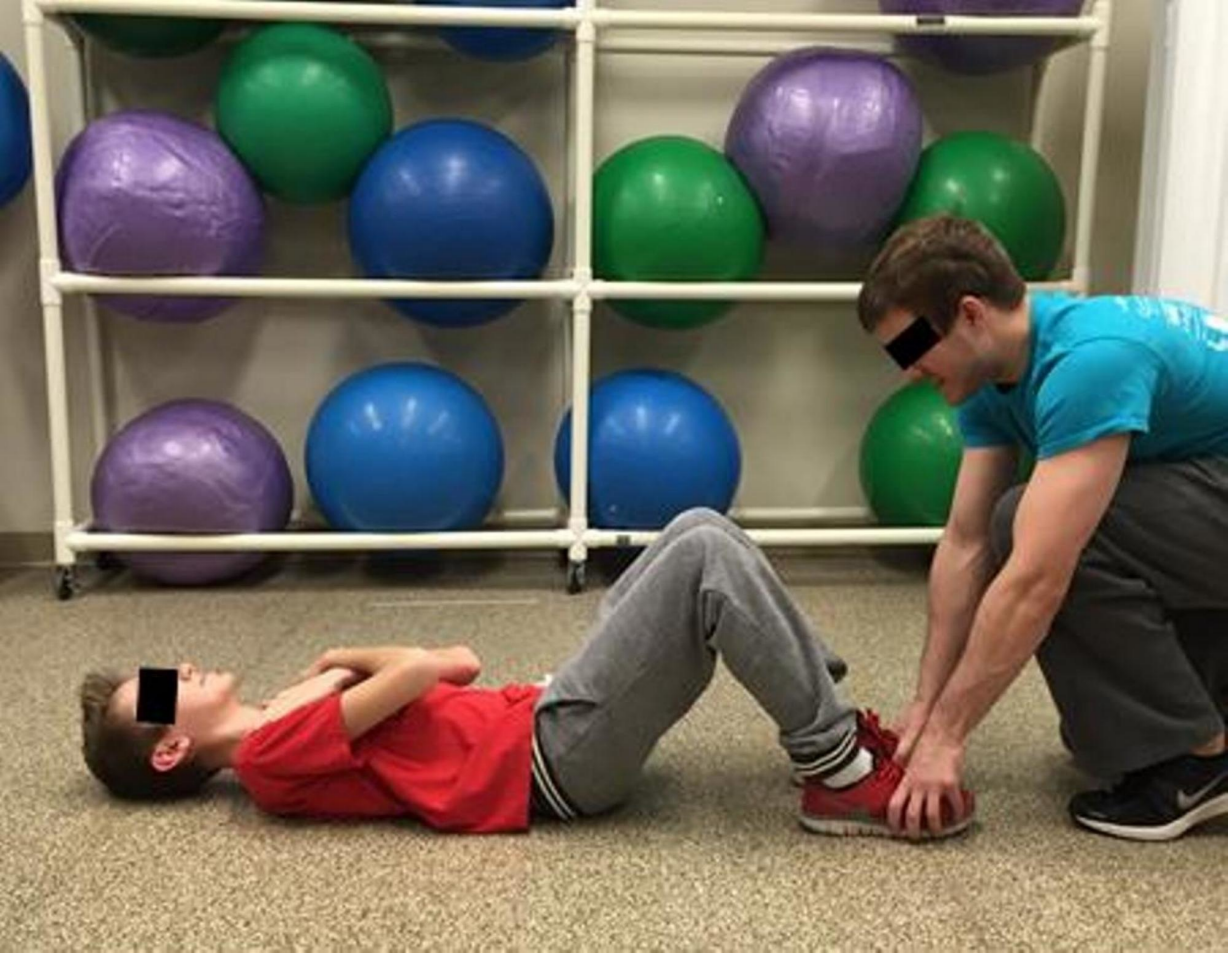
Sit-up start and end position

**Figure 8:**
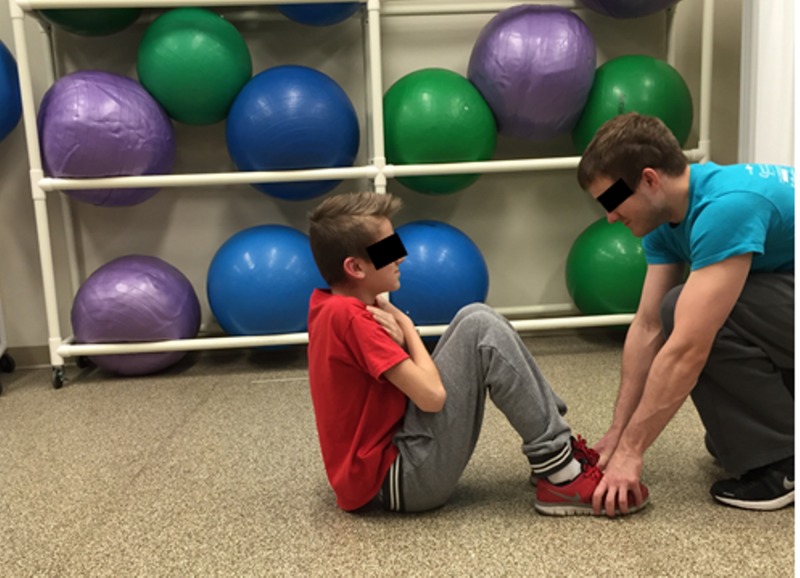
Sit-up mid-test position

The test began in supine position, knees flexed, feet flat on the floor, and heels 12 to 18 inches away from the buttocks. The arms were crossed on the chest with hands on opposite shoulders and the feet were held by the investigator to assure that the feet remained flat on the floor (Figure 7) as is the required measurement protocol of the PFMB [5]. The scout then sat up by tucking the chin to the chest with arms remaining on the chest and curled up to sitting position until the elbows touched the thighs (Figure 8). The scout returned to the starting position with shoulder blades touching the floor and repeated the process as many times as possible in 60 seconds.

One Mile Run Test

The 1MR was performed on an indoor track with a pre-established distance formula based on the lane used and number of laps required for covering a distance of one mile. Each scout was instructed to cover the distance as quickly as possible, but walking was permitted. The investigator used a stopwatch and lap counter to record the time to complete one mile in minutes and seconds.

Non-merit badge required fitness tests

Grip, Tip Pinch, and Key Pinch Tests

Performance of the grip, tip pinch, and key pinch tests used a previously established protocol [10]. All measurements were obtained with the scout seated in a chair, shoulder adducted and neutrally rotated, elbow flexed to 90°, and the forearm and wrist in a neutral position.

Grip strength was measured using the Baseline Hydraulic Hand Dynamometer (Fabrication Enterprises, Inc., White Plains, NY). The dynamometer was placed against the metacarpals while the phalanges wrap around the device. When cued by the recorder, the scout gripped the dynamometer as forcefully as able. Two grips were allowed with the scout’s dominant hand and the recorder took the highest measurement overall.

Tip pinch and key pinch were measured using the Jamar Hydraulic Pinch Gauge (Sammons Preston Rolyan, Bolingbrook, IL). For the tip pinch test, the measurement device was placed between the thumb and tip of the index finger in the dominant hand and the scout was instructed to pinch as forcefully as able with the index finger and thumb. For the key pinch test, the measurement device was placed between the thumb pad and lateral aspect of index finger in the dominant hand and the scout was instructed to pinch as forcefully as able. For both the tip pinch and key pinch tests, two measurements were obtained on the dominant hand and the highest of the measurements was recorded.

Illinois Agility T-test

**Figure 9:**
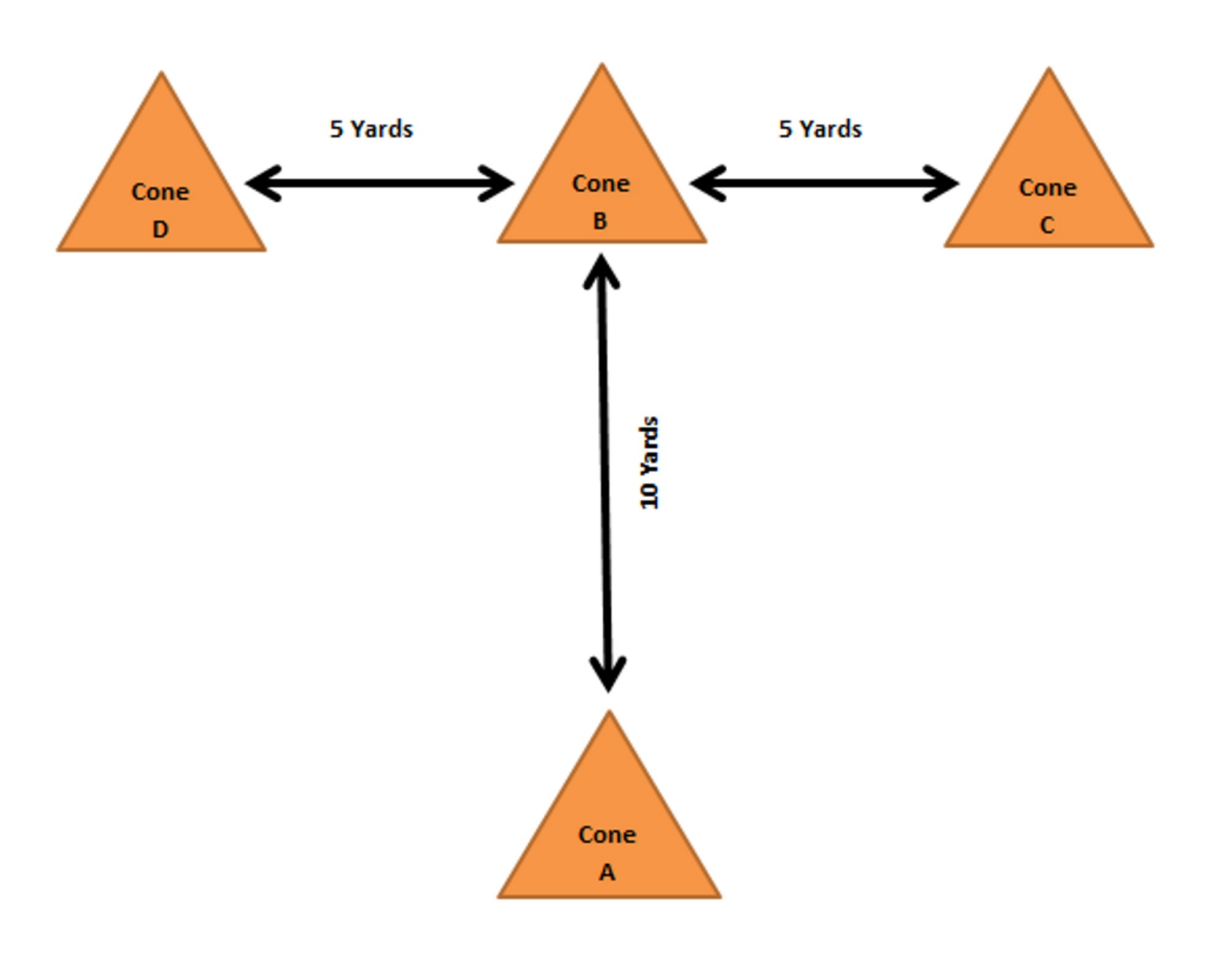
Agility T-test protocol

Illinois Agility T-testing methods utilized a previously established protocol [11]. Scouts began the test at a starting line marked with a cone (Cone A) in a staggered stance. Three additional cones were placed at 10 yards (9.14 meters) in front of the starting line (Cone B) and two at 5 yards (4.57 meters) perpendicular to either side of Cone B (Cones C and D). Upon initiation of a timer, scouts sprinted to Cone B and touched it with right hand, shuffling right to Cone C and touched it with right hand, shuffled left to Cone D and touched with left hand, shuffled back to Cone B and touching with left hand and finally ran backwards to the start line (Cone A). This protocol is diagrammed in Figure 9. The time from leaving the starting line to return to the starting line was recorded in minutes and seconds.

Vertical Jump Test

This FT was modified from the original Sargent Jump [12] and performed as follows: 1) a tape measure with a length of 0-100 cm was positioned vertical to the floor and secured on a wall 150 cm from the floor, 2) the scout stood behind a line positioned one foot from the wall with heels flat, feet shoulder-width apart, and arms outstretched overhead with hands overlapping, 3) a baseline measurement was established by having the scout reach as high above their head as possible and note the height of their reach on the tape measure, and then 4) a jump maneuver was performed by squatting to a self-selected depth with arms behind his body, pausing 1-3 seconds, and then jumping as high as able. The investigator used the best of three jumps to establish the vertical jump height.

Data Analysis

Descriptive statistics were determined for age, BMI, BMI category, and the results of each FT. A Kruskal-Wallis test, a nonparametric test used when the assumptions of one-way ANOVA are not met, examined pairwise comparisons between BMI categories and each FT with significance set at p<0.05. A nonparametric Spearman's correlation examined the correlations between performance of each FT to both age and BMI. Correlations were determined to be significant with a p-value < 0.05. Statistical analysis was performed using SAS v.9.4 software for Windows (SAS Institute Inc., NC, USA).

## Results

Demographics

A total of 100 scouts presented to the PFMB event. One declined to assent and so he was excluded. This resulted in 99 Boy Scouts aged 11-17 (mean = 12.61 (SD=1.41)) meeting the study inclusion criteria. Thirty-three were enrolled in each of the three data collection years.

Body mass index

The mean height of participants was 63.43 inches (SD=4.3) or 161.11 cm (SD=11.0); whereas, the mean weight was 125.8 lbs. (SD=42.8) or 57.2 kg (SD=19.4). The mean BMI was 21.5 kg/m^2^ (SD=5.4) with category frequencies as follows: underweight (n=8), normal weight (n=56), overweight (n=14), and obese (n= 21).

Description of fitness test

Table 1 provides the results of both the PFMB and non-merit badge FTs. It is notable that the indoor track became unavailable during the data collection period. Therefore only 83 scouts completed the 1MR testing making the sample size for analysis smaller than expected.

**Table 1:** Descriptive statistics of all fitness tests

VARIABLE	n	Mean (SD)	Range
Merit Badge Required Fitness Tests			
Sit and Reach Flexibility Test (cm)	98	21.2 (10.5)	(-2) - 40
Push-ups	99	18.0 (10.6)	0-53
Pull-ups	99	1.8 (2.7)	0-10
Sit-ups	99	28.9 (8.7)	9-52
One Mile Run (seconds)	83	616.6 (156.8)	362-1091
Non-Merit Badge Required Fitness Tests			
Grip Test (lbs.)	98	50.5 (17.4)	24-105
Tip Pinch Test (lbs.)	98	10.9 (3.2)	5-20
Key Pinch (lbs.)	97	15.7 (3.5)	10-29
Illinois Agility T-test (seconds)	98	14.8 (2.4)	10-21.2
Vertical Jump (cm)	94	33.5 (10.0)	12-66
cm = centimeters, lbs. = pounds			

Body mass index category comparison to fitness test performance

Table 2 reports the FT comparisons across BMI categories and identified significance among the following FT: push-ups, pull-ups, sit-ups, 1MR, and tip pinch. Table 3 then reports the pairwise comparisons of the BMI category for the significant FT comparisons identified in Table 2. Statistically, lower performance was identified in the PFMB required FT when comparing overweight and obese categories to the normal weight category for push-ups, pull-ups, sit-ups, and 1MR; whereas, the underweight category performed better than the obese category pull-ups and sit-ups. Additionally, the overweight category performed better on the tip pinch test compared to both the normal and underweight categories. While the obese category outperformed the underweight category in the tip pinch, this finding did not meet a level of significance (p=0.05).

**Table 2:** Fitness test performance compared to body mass index categories

	Underweight		Normal		Overweight		Obese		
VARIABLE	n	Mean (SD)	n	Mean (SD)	n	Mean (SD)	n	Mean (SD)	p-value
Merit Badge Required Fitness Tests									
Pull-ups	8	2.5 (2.8)	56	2.5 (2.9)	14	0.2 (0.4)	21	0.6 (2.2)	0.001
Push-ups	8	19.6 (11.6)	56	20.4 (10.1)	14	12.6 (10.1)	21	14.6 (10.6)	0.04
Sit and Reach Flexibility Test (cm)	8	15.9 (8.9)	55	21.8 (11.1)	14	19.0 (11.0)	21	23.0 (8.7)	0.26
Sit-ups	8	31.6 (7.3)	56	31.3 (8.4)	14	24.9 (6.3)	21	24.0 (8.7)	0.003
One mile run (seconds)	7	612.3 (196.2)	46	544.7 (114.1)	12	670.9 (114.8)	18	755.1 (161.6)	0.001
Non-Merit Badge Required Fitness Tests									
Grip test (lbs.)	8	40.5 (11.6)	56	51.0 (17.3)	13	58.5 (23.0)	21	48.0 (4.2)	0.19
Tip pinch test (lbs.)	8	8.6 (2.0)	56	10.7 (3.1)	13	12.6 (3.5)	21	11.0 (3.0)	0.03
Key pinch (lbs.)	8	14.1 (2.5)	56	15.6 (3.4)	13	17.2 (4.9)	20	16.4 (2.8)	0.14
Illinois Agility T-test (seconds)	8	13.8 (1.1)	55	14.5 (2.4)	14	15.4 (2.0)	21	15.6 (2.9)	0.08
Vertical Jump (cm)	8	30.7 (10.6)	53	35.9 (11.0)	14	30.4 (7.3)	19	30.2 (6.7)	0.12
cm = centimeters, lbs. = pounds									

**Table 3:** Pairwise comparison of body mass index categories for fitness tests identified as significant in Table 2

VARIABLE	underweight versus normal weight	underweight versus overweight	underweight versus obese	normal weight versus overweight	normal weight versus obese	overweight versus obese
	p-values					
	Merit Badge Required Fitness Tests					
Push-ups	0.73	0.29	0.24	0.02	0.03	0.61
Pull-ups	0.99	0.07	0.02	0.002	0.001	0.42
Sit-ups	0.65	0.06	0.03	0.01	0.003	0.77
One Mile Run	0.73	0.31	0.08	0.001	0.001	0.16
	Non-Merit Badge Required Fitness Tests					
Tip pinch	0.08	0.02	0.05	0.03	0.53	0.16

Fitness test correlations to age and body mass index

Table 4 reports the results of the correlation analysis of the PFMB and non-merit badge FT to age or BMI. While no correlations between advancing age and improved FT performance were identified in the PFMB required FT, correlations were observed for the grip, tip pinch, key pinch, vertical jump, and T-test. Furthermore, correlations between a higher BMI and lower FT performance were identified for the pull-up, sit-up, and 1MR; whereas, a higher BMI correlated to more positive test performance on the grip, key, and tip pinch tests.

**Table 4:** Fitness test correlations to age and body mass index

VARIABLE	Correlation with Age (R value)	p-value	Correlation with BMI (R-value)	p-value
Merit Badge Required Fitness Tests				
Sit and Reach Flexibility Test	0.07	0.52	0.18	0.07
Push-ups	0.07	0.52	-0.17	0.10
Pull-ups	0.02	0.87	-0.26	0.01
Sit-ups	-0.03	0.80	-0.36	0.001
One Mile Run	-0.14	0.22	0.39	0.001
Non-Merit Badge Required Fitness Tests				
Grip Test	0.50	0.001	0.27	0.01
Tip Pinch Test	0.33	0.001	0.32	0.002
Key Pinch	0.40	0.001	0.41	0.001
Illinois Agility T-test	-0.31	0.002	0.14	0.18
Vertical Jump	0.25	0.01	-0.09	0.40
BMI= Body Mass Index				

## Discussion

The purpose of this study was to describe and correlate FT to age and BMI among adolescent Boy Scouts. Variation in a scout’s FT performance is evidenced in the large range of FT outcomes for the PFMB required FTs as well as the non-merit badge required FTs. Evidence for causation of the breadth of performance levels may be further explained by examining the age and BMI of the participating scouts.

While prior evidence has established a relationship between fitness and age [13-15], no significant relationships were observed between age and the five PFMB FTs. However, significantly improved performance differences were identified in older scouts in each of the non-merit badge required FTs. This supports the continued inclusion of the current PFMB FTs as variability in the age of scouts (11-17 years) embarking on the PFMB requirements would not inherently bias a scout’s ability to have positive outcomes on the initial FT measures.

A scout's BMI category provides significant causation for variation in FT outcomes. Specifically, this study identified that scouts in the normal BMI range outperformed their overweight and obese counterparts in all PFMB required FT with the exception of the SRF. It is unknown if weight control interventions were previously or retrospectively implemented for participating scouts at risk for becoming obese or meeting the obese criterion. However, it seems plausible that the physician conducting the PFMB required participation medical clearance or other healthcare providers, inclusive of physical therapists, could include strategies such as motivational interviewing or referrals to registered dietitians or health coaches into their care plans in an effort toward optimization of a scout’s BMI. It is notable that the tip pinch test, a non-merit badge FT, had more favorable outcomes among higher weight scouts, which may warrant further investigation.

A prior study has reported that participation in the required activities of the PFMB demonstrated an improvement on cardiovascular endurance, specifically VO2, among 14 scouts [16]. The authors of that study concluded that the PFMB is beneficial as a physical activity intervention [16]. Therefore, the PFMB may prove to be another useful tool in addition to the Centers for Disease Control and Prevention-Youth Physical Activity Kit [17] and the Presidential Youth Fitness Program [18]. Each may assist to guide adolescents toward recommended physical activity and fitness levels. Furthermore, the BSA PFMB could be considered as an adjunctive tool for use in non-scouting adolescents; however, further research is warranted to investigate this generalization.

The current PFMB book [5] does provide evidence-based recommendations for a scout’s optimal nutrition. However, expansion of the PFMB requirements to include a caloric intake/output self-assessment activity may be beneficial in offering scouts insight into their caloric consumption and expenditure related behaviors. This may be of greatest benefit to scouts in the overweight and obese categories to establish awareness of caloric imbalances of which a scout may not be cognizant.

While the PFMB requires scouts to perform either a pull-up or push-up FT as a component of initial testing and re-testing, it may be beneficial for scouts to conduct both FTs. Different muscle groups are involved with each activity and the exercises correspond to different scouting activities, such as rock climbing and rappelling (pull-up) or pushing camping equipment or a canoe into the water (push-up). As 55 of the scouts were unable to perform even one pull-up, inclusion of an option for scouts unable to perform a standard pull-up, such as a modified pull-up [19] is warranted. The modified pull up technique uses altered biomechanics with the main advantage for scouts unable to perform the standard technique being that their entire body weight is not being pulled against gravity. This would be advantageous to scouts seeking to make strength gains to the specific muscle groups required for a pull-up and provide training benefits toward ultimate achievement of the standard pull-up over time.

Study limitations

Although DPT students received pre-training of FT measurement and oversight was provided by licensed physical therapists, the potential for decreased reliability in the FT results is plausible considering the number of DPT student data collectors and limited prior experience with the assessment tools employed. Additionally, a sample size that varied by age, specifically, smaller sample sizes with increasing age, and a time lapse between the three data collection years may result in a type II error for age comparisons and correlations. Furthermore, use of a sample of convenience may have included only scouts interested in earning the PFMB and limits generalizability to all Boy Scouts. Finally, a scout’s effort on each FT may have impacted measurement outcomes.

Future research

Future research examining changes in FT measures upon completion of a scout's self-determined 12-week exercise program to establish efficacy in bringing about change in fitness is warranted. Additionally, examination of the types and amounts of exercise documented by the scouts who complete this merit badge may add insight into future educational strategies to assure that scouts understand the national daily physical activity recommendations to optimize health and fitness for adolescents. Furthermore, examination of the existence of an optimal time for scouts to initiate the PFMB, potentially using the Transtheoretical Model [20], to determine a scout’s readiness for change may be useful. Establishing normative values for scouts of varied age and weight category may be of benefit. Lastly, as the BSA has now opened eligibility to register as a scout to females, an examination of these measures to include female scouts is warranted.

## Conclusions

Variations in FT performance levels were observed among Boy Scouts completing the initial FT requirements of the PFMB. While prior evidence suggests improved FT scores with advancing age in adolescent males, this was not observed; however, less favorable performance on the PFMB required FT was detected among overweight and obese scouts.
